# Malocclusion and Its Relationship with Sound Speech Disorders in Deciduous and Mixed Dentition: A Scoping Review

**DOI:** 10.3390/dj13010027

**Published:** 2025-01-10

**Authors:** Mariangela Aprile, Alessio Verdecchia, Claudia Dettori, Enrico Spinas

**Affiliations:** 1 Department of Surgical Sciences, Postgraduate School in Orthodontics, University of Cagliari, 09124 Cagliari, Italy; mariangela.aprile@hotmail.it; 2 Department of Surgical Sciences, School of Dental Medicine, University of Cagliari, 09124 Cagliari, Italy; claudia.dettori@unica.it

**Keywords:** deciduous dentition, dyslalias, malocclusion, mixed dentition, pediatric patients, speech sound disorder

## Abstract

**Objectives:** The intricate relationship between malocclusions and speech sound disorders (SSDs) is yet to be fully understood. This is particularly true for pediatric patients during the deciduous and mixed dentition stages. Employing a methodical scoping review approach, this study scrutinizes the recent literature to elucidate how these dental misalignments impact speech articulation and phonetic clarity. **Methods:** The present scoping review has been conducted following the Preferred Reporting Items for Systematic reviews and Meta-Analyses extension for Scoping Reviews (PRISMA-ScR) guidelines. The selected articles have been found using PubMed, Scopus, Web of Science, and The Cochrane Library; the scope was limited to studies describing cases of patients in the deciduous or mixed dentition stage and the presence of both malocclusion and SSDs. **Results:** Out of the 1880 articles found, 44 passed the initial screening and 12 met the eligibility criteria and have been included in this review. **Conclusions:** The analysis reveals that while there is a consensus on the influence of malocclusions on speech production, the extent and specific nature of these effects vary across studies. anterior open bite is frequently associated with speech disorders, affecting phonemes by altering airflow and tongue placement. The review highlights the need for multidisciplinary approaches for effective treatment and calls for further investigation into the causative relationships between malocclusions and SSDs.

## 1. Introduction

The term malocclusion refers to an abnormal alignment of the teeth and a discrepancy in the way the upper and lower teeth fit together. This definition encompasses a range of issues, from simple crowding of teeth to more complex problems involving the growth and development of the jaws [1]. Malocclusions are a common dental condition in childhood and adolescence. They are considered a worldwide health problem, with diverse manifestations across different populations and are caused by genetic and environmental factors [2]. These factors include growth patterns, muscle functions, breathing patterns, and early tooth extractions or losses [3,4]. Moreover, the modeling forces of the muscles over the dental arches are disrupted by harmful oral habits including non-nutritive sucking (NNS) habits and residual orofacial dysfunctions like incorrect tongue position and open-mouth posture. These factors are especially impactful in the primary and mixed dentition stages and are commonly declining from the ages of 3 to 12 years [4]. A notable example is the anterior open bite (AOB), a manifestation of malocclusion whose etiology remains uncertain, but is believed to be multifactorial and influenced by variations in dental eruption or alveolar growth, disproportionate neuromuscular growth, and tongue malfunction [5].

Malocclusions are often associated with atypical swallowing, a non-physiologic condition characterized by the persistence of infantile swallowing patterns even after the permanent dentition is in place. The causative and resultant roles of these two conditions are still unclear [5,6], given that certain oral habits and tongue protrusion can exacerbate malocclusion [7,8,9]. Orofacial dysfunction might also influence occlusal development [3,10] and an association has been observed between atypical swallowing patterns and relapse after orthognathic surgery [11].

The literature indicates that malocclusion transcends beyond aesthetic appearance, significantly impacting oral health and functionality [4]. In school children, it can lead to non-aesthetic traits, poor lingual position, and consequently, changes in speech, potentially affecting the quality of life [12,13]. There is evidence of the association of certain swallowing patterns, particularly those involving tongue interposition, with “lisping”—an altered production of sibilant sounds [5,6].

Speech is an intricate process involving the planning and execution of precise neuromuscular sequences and is profoundly impacted by the structural dynamics of the oral cavity. The causal relationship between speech disorders and oral anomalies is complex, often tied to the anatomical structure of the oral cavity, but also potentially influenced by language-specific characteristics [14]. This complexity is exemplified in the relationship between malocclusion and tongue position, which the Balance Theory helps to elucidate, indicating that correctly positioned teeth aid in balancing the forces between the tongue and labio-buccal muscles [15]. Speech is usually impaired when the function of any of the components involved in the articulation of phonemes is abnormal. Respiratory disorders, laryngeal abnormalities, and alterations of articulation organs may cause issues such as stuttering, hoarseness, and dyslalias (a disorder in the articulation of phonemes, altering, omitting, or replacing sounds incorrectly).

Malocclusions are intrinsically linked to speech difficulties [1], but their severity is not always proportional to the severity of the malocclusion [16]. Speech sound disorders are common in developing children and considered neurodevelopmental disorders (NDDs) [17,18,19]. The etiology can range from idiopathic to genetic or acquired diagnoses [19], with structural deviations in the oral cavity, particularly in the anterior part, significantly affecting lip and tongue placement for speech sounds [20,21,22]. AOB stands out as the most prevalent malocclusion associated with speech disorders [23], especially in pediatric patients [18,19,24,25,26,27].

Finally, the work of researchers such as Fymbo [25,26], Snow [28], and Bloomer [29], summarized by Johnson in his review [23], has been pivotal in exploring the relationship between malocclusions and oral cavity anomalies. Their findings affirm that the integrity of dental arches is crucial for correct speech production, emphasizing the considerable influence of both upper and lower arches on speech articulation and the evident correlation between malocclusion and speech difficulties.

The goal of this scoping review is to investigate evidence on the causal relationship between malocclusions and SSDs in deciduous and mixed dentition stages. As a secondary objective, we will explore which malocclusions have a larger impact on speech production.

## 2. Materials and Methods

This scoping review was conducted following the “Preferred Reporting Items for Systematic Reviews and Meta-Analyzes extension for conducting Scoping Reviews” (PRISMA-ScR) [30] guidelines. This scoping review was not registered. In order to specify the research strategy parameters, the primary research question has been stated as “Is there a causal relationship between malocclusion and Speech Sound Disorders in pediatric patients without other contributing syndromes?”.

The electronic search was conducted via PubMed, Scopus, Web of Science, The Cochrane Library, and OpenGrey for grey literature on 16 December 2024, by means of the search strategy shown in Table 1.

The corpus was screened for inclusion using the listed criteria, explicitly following the PICO format:

- Population: Pediatric patients in deciduous or mixed dentition without syndromes.
- Intervention: Studies investigating speech sound disorders.
- Comparison: Studies exploring malocclusions.
- Outcomes: Studies assessing the impact of different malocclusions on speech sound disorders.

The inclusion and exclusion criteria that were applied are summarized in Table 2.

The search strategy for this scoping review involved a thorough manual examination of the reference lists of all articles initially retrieved, to ensure a comprehensive review of relevant literature. There were no limitations to the publication year or language.

The review focused exclusively on studies involving human subjects.

To streamline the process, all references were uploaded to the Rayyan^®^ application (accessed on 16 December 2024), which was also used to eliminate duplicate entries. Following this, titles and abstracts were reviewed, and articles deemed pertinent were then subjected to a full-text review to determine their suitability for inclusion in the review. This screening of abstracts and full-text manuscripts was conducted by two reviewers independently (MA and AV) to ensure objectivity and thoroughness in the selection process. In cases of disagreement, a third reviewer (ES) was involved to make a final decision whether to include an article or not.

The collected information for each study was organized into a table format with the data collected from each study organized in the following key areas: author, year of publication, setting and location of the study, the number of participants in each study, the age range of the participants, the design of the study, and the results obtained from each study. Moreover, for each study included in this review, the authors kept track of the malocclusions considered and which phonemes were related to each malocclusion. This structured approach to data collection and organization facilitated a clear and comprehensive synthesis of the research findings.

## 3. Results

### 3.1. Search Details

**Figure 1:**
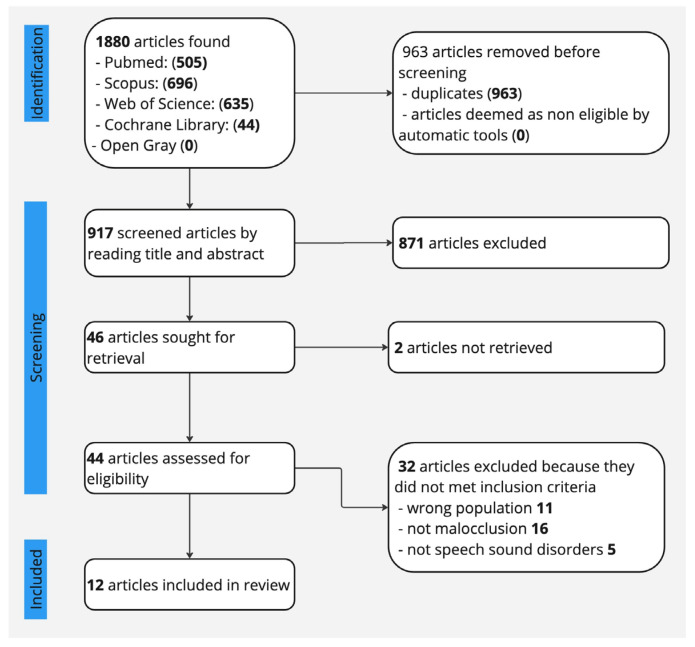
Flow diagram of study selection.

The described search gave 1880 articles as a result: 505 from Pubmed, 696 from Scopus, 635 from Web of Science, 44 from The Cochrane Library, and 0 from Opengrey. An initial screening was conducted in order to remove the 963 duplicates. After titles and abstracts were read, 46 articles remained for further evaluation. The remaining articles were thoroughly analyzed by a full-text reading, and 12 studies were considered eligible for inclusion in this review (see Figure 1). Characteristics of the included studies are shown in Table 3. We used Retraction Watch Database to verify that at the time of writing, none of the eligible studies had been retracted.

### 3.2. Study Characteristics

The study characteristics are summarized in Table 3, specifically, author, year of publication, setting, country, sample size, age range, and study result. All the included studies are cross-sectional studies. Only three studies compared the results against a control group [31,32,33].

The most represented country was Brazil with five studies included [34,35,36,37,38], followed by Sweden with two [32,33], while only one article was included for Poland [39], Italy [31], USA [40], Spain [41], and Colombia [42].

The population size of the various studies ranged from 69 to 880 children; only 1 study [38] recruited less than 100 children, while 5 out of 12 recruited at least 200 children [31,34,36,40,41].

Participant ages ranged from 3 to 16 years, with most studies considered patients in the mixed dentition stage, while only two [36,38] included only patients in deciduous dentition.

The studies included in this review show higher prevalence, type, and severity of malocclusions in children with speech sound disorders compared to those with typical speech development [32,39]. Studies indicate a strong correlation between 150 malocclusions and SSDs, suggesting that malocclusions, especially those affecting tongue position, may act as risk factors for SSDs [31,34,39,41,42]. Specifically, AOB is frequently associated with anterior tongue thrust, lip incompetence, and anterior lisping, particularly for lingua-alveolar sounds [35,36,38]. Additionally, children with SSDs and poorer orofacial function are more likely to present with malocclusions [33].

**Table 3:** Table summarizing author, year of publication, setting and location, population size, age range, and results of the eligible articles. All the listed articles follow a cross-sectional study design.

Article	Setting	Sample Size	Age Range (in Years)	Result
Mogren et al., 2022 [32]	Sweden	105	6–16	Higher prevalence, type, and severity of malocclusions in children with SSDs compared to a control group of children with typical TSD
Grudziąż-Sękowska et al., 2018 [39]	Poland	161	7–10	Strong correlation between malocclusion and SSDs. The prevalence of malocclusions was higher in the group with SSDs compared to the group without SSDs
Assaf et al., 2021 [34]	Federal University of Santa Maria/Brazil	547	7–13	Relationship between malocclusion and SSDs. Findings suggest that malocclusion is a risk factor for SSDs, particularly in cases where tongue position is affected
Farronato et al., 2012 [31]	University of Milan Fondazione IRCCS Cà Granda—Ospedale Maggiore Policlinico/Italy	880	6–10	Strong statistical correlation between malocclusions and dyslalias
Amr-Rey et al., 2022 [41]	Dentistry and Medicine Faculty, University of Valencia/Spain	290	4–10	Strong relationship between malocclusion and speech disorders. Both may be associated with oral habits and muscular alterations, causing orofacial dysfunction
Maciel et al., 2005 [35]	Federal University of Juiz de Fora—University Hospital/Brazil	130	8–12	A correlation between the etiology of anterior open bite, deleterious oral habits, and a few orofacial malfunctions. An association between the history of deleterious habits and the occurrence of lingual interposition during swallowing as well as SSDs was identified
Sahad et al., 2008 [36]	Department of Orthodontics, University of São Paulo City/Brazil	333	3–6	Significant relationship between open bite and anterior lisping and/or anterior tongue thrust in the articulation of the linguo-alveolar phonemes. Significant relationship between deep over-bite and the absence of anterior lisping and anterior tongue thrust in the articulation of the linguo-alveolar phonemes; No significant relationship between normal over-bite or edge-to-edge occlusion and phonoarticulatory occurrences
Farret et al., 1998 [37]	Federal University of Santa Maria/Brazil	113	9–14	Correlations between malocclusion and articulatory problems. Significant relationship between malocclusion and speech disorders in all the angle classifications. In Class III, the results were highly significant
Wadsworth et al., 1998 [40]	California State University/USA	200	5–12	Absence of statistically significant relationships between dental malocclusions and dentalization of phonemes. However, after increasing the alpha level to *p* < 0.05, dental malocclusion was found to be significantly related to the dentalization of phonemes
Mogren et al., 2022b [33]	Sweden	105	6–16	Among children with SSDs, those with poorer orofacial function were more likely to have a malocclusion
Verrastro et al., 2006 [38]	University of São Paulo School of Dentistry/Brazil	69	3–5	The orofacial myofunctional characteristics associated with AOB were anterior tongue interposition during swallowing and speech and lip incompetence.
Ocampo-Parra et al., 2015 [42]	Universidad Cooperativa de Colombia/Colombia	132	8–16	Relationship between malocclusion and SSDs, particularly with AOB

Abbreviations: SSD, speech sound disorders; TSD, typical speech development; AOB, anterior open bite.

The studies included in this review show higher prevalence, type, and severity of malocclusions in children with SSDs compared to those with typical speech development (TSD) [32,39]. Studies indicate a strong correlation between malocclusions and SSDs, suggesting that malocclusions, especially those affecting tongue position, may act as risk factors for SSDs [31,34,39,41,42]. Specifically, AOB is frequently associated with anterior tongue thrust, lip incompetence, and anterior lisping, particularly for linguo-alveolar sounds [35,36,38]. Additionally, children with SSDs and poorer orofacial function are more likely to present with malocclusions [33].

Other studies have identified significant correlations between different types of malocclusions and articulatory problems, with particularly strong correlations noted in Class II and Class III cases [37]. One of the studies [40] did not find a significant link between malocclusions and phoneme distortions until adjustments in the statistical thresholds revealed the relationship between malocclusion and phoneme dentalization.

### 3.3. Dento-Skeletal Characteristics

Each study included in this review approached the correlation between malocclusions and SSDs in slightly different ways; hence, not all studies considered every possible malocclusion. Table 4 summarizes which malocclusions have been investigated in which study.

The most prevalent malocclusion was the AOB, which was considered by 10 articles [31,32,33,34,35,36,38,40,41,42], followed by deep bite [31,32,33,34,36,39,40,41]. The studies that considered Class II malocclusions also considered Class III malocclusions [31,32,33,34,37,39,40,41], while patients with overjet were included in [31,34,36,40,41], and [31,34,40,41] had patients with edge-to-edge bite.

The least represented dento-skeletal characteristics are Class I malocclusions [31,34,37], crowding [32,39], and scissor bite [31].

**Table 4:** Malocclusions taken into consideration by each article.

Article	AOB	I Class	II Class	III Class	Cross-Bite	Deep Bite	Overjet	Edge-to-Edge Bite	Scissor Bite	Crowding
Mogren et al., 2022 [32]	x		x	x	x	x				x
Grudziąż-Sękowska et al., 2018 [39]			x	x	x	x				x
Assaf et al., 2021 [34]	x	x	x	x	x	x	x	x		
Farronato et al., 2012 [31]	x	x	x	x	x	x	x	x	x	
Amr-Rey et al., 2022 [41]	x		x	x	x	x	x	x		
Maciel et al., 2005 [35]	x									
Sahad et al., 2008 [36]	x					x	x			
Farret et al., 1998 [37]		x	x	x						
Wadsworth et al., 1998 [40]	x		x	x		x	x	x		
Mogren et al., 2022b [33]	x		x	x	x	x				
Verrastro et al., 2006 [38]	x									
Ocampo-Parra et al., 2015 [42]	x									

Abbreviations: AOB, anterior open bite. “x” indicates if the study assessed population for that malocclusion.

### 3.4. Phonemes and Their Association with Malocclusions

Although all studies included in this review investigated the correlation between malocclusions and SSDs, some studies [31,36,37,39,40,41,42] granularly investigated the correlation between each malocclusion and phonation defects on specific phonemes.

Table 5 shows each phoneme considered in the studies included in this review and which malocclusion it has been associated with and by which studies. Given that the primary language of the population varies across studies, not all studies considered all phonemes presented in the table, and some phonemes are present only in certain studies. A notable example is [39], which included typical phonemes of the Polish language that have not been considered by other studies. Table 6 provides an explanation of the phoneme categorization that is used across the discussion; refer to [43] for a complete reference.

The most impacting malocclusion is again the AOB, which has been reported to impact sibilant (/s/, /z/ and /ch/) sounds by [37,39,40,41,42], linguo-alveolar sounds (/t/, /d/, /n/, /l/) by [36,39,40,41,42], fricatives and affricates (/f/, /v/, /ȝ/) by [37], and lateral sounds (/l/, /ll/) by [36,41].

The impact of Class II malocclusion and Class III malocclusion seems to be largely similar, being reported to have a significant relationship with mispronunciation of rhotic sounds (/r/, /rr/) by [37,41], sibilants (/s/, /z/ and /ch/) by [37,39,41], fricatives and affricates (/f/, /v/, /ȝ/) by [37,41], and lateral sounds (/l/) by [40]. The most notable difference is that linguo-alveolar sounds (/t/, /d/, /n/, /l/) have been found to be related to Class II malocclusion by [39,40] only, while [37,39,40,41] found correlations with Class III malocclusion.

While cross-bite has been encountered by half of the studies included in this review (see Table 4), it has only two studies [37,41] have found significant correlation with the mispronunciation of rhotic sounds (/r/, /rr/), sibilant (/s/, /z/ and /ch/), fricatives and affricates (/f/, /v/, /ȝ/) and linguo-alveolar sounds (/t/, /d/, /n/, /l/).

Edge-to-edge bite has been related to fewer phonemes than the other malocclusions considered. Only phonemes (/r/, /s/, /z/, /t/,/l/) from the previously listed phoneme groups have been reported by [41] and only the phoneme /t/ has been found to be related to edge-to-edge bite by [37].

The Polish-specific fricatives and affricates (/ɕ/, /ʑ/, /t͡s/, /d͡z/, /t͡ɕ/, /d͡ʑ/) were only considered by [39] and related to Class II and III malocclusion and AOB (with the exclusion of /ɕ/ and /ʑ/).

## 4. Discussion

The studies included in this scoping review found a significant correlation between different malocclusions and speech sound disorders, impacting the pronunciation of a wide selection of phonemes. The most cited and studied malocclusion is the AOB, which is strongly related to SSDs as well as abnormal tongue position at rest, tongue interposition, and tongue thrust swallowing.

In pediatric age, during the stages of primary and mixed dentition, it is common to observe the presence of malocclusions with a peak at 3 years of age [7,33,44]. These do not appear in isolation but are often associated with a range of external factors that influence the development and positioning of oral structures. Among these are non-nutritive sucking practices, such as thumb sucking and prolonged pacifier use, open-mouth posture, abnormal tongue position at rest, tongue interposition during swallowing, tongue thrust swallowing, and other parafunctional habits. If left unresolved, all these factors tend to negatively impact the orofacial complex, altering tooth positioning, jaw development, and the balance of muscular forces [33].

It is interesting to note that these parafunctional habits tend to diminish naturally toward the end of the mixed dentition phase, as children reach a level of physical and behavioral maturity. In parallel, malocclusions also tend to decrease during this period, but only if there is a concurrent resolution of the aforementioned factors. If these habits persist, however, there is a risk that the malocclusion will become permanent [4,44,45,46]. Sucking habits during primary dentition can also cause malocclusions in later development stages [7,44].

Some authors [36,47] suggest that the structure of the orofacial complex can influence function, and that the function can influence occlusion, dental positioning, and jaw structure and development. Speech sound disorders are likely another dimension within this multifactorial context. Following Dimberg’s conclusion [7] that orthodontic treatment of malocclusions diagnosed during primary dentition should be deferred until mixed dentition, an early functional treatment, including speech therapy, might help reduce the need for orthodontic treatment. A similar therapeutic approach should be adopted in cases of early loss of deciduous incisors, as this condition may act as a predisposing factor for the development of SSDs [3].

There is some disagreement in the literature about the impact of tongue movements and tongue position at rest on jaw development. Some authors deem the lasting and continuous effect of a misplaced tongue at rest more important for jaw development than its movements during chewing, swallowing, and speaking [44]. Contrariwise, some authors consider the influences of muscle forces during speaking, articulation, and swallowing to be more impactful on the orofacial complex, even if their nature is intermittent [36]. The causal relationship between malocclusion, tongue position, and speech defects is not clear and cannot be proven [31,33,34], even with a number of studies proving the correlation between these aspects [48,49,50].

Spoken language requires a complex coordination of the tongue, lips, and other vocal apparatus, as it relies on their ability to manipulate the airstream produced by the lungs [23,33]. Speech sounds are categorized into vowels and consonants. While the former are produced by vibrating the vocal cords without obstruction of the air stream, the latter require the interference of the lips, tongue, teeth, palate, and nose [51]. Here is the reason why any modification of the orofacial complex can potentially impact the speech sound production of consonants.

Phonemes in the same category might be affected by similar issues, e.g., sibilant sounds (/s/, /z/, and /ch/) are produced with the tip of the tongue close to the alveolar ridge, just behind the upper teeth, keeping the teeth close together without touching in order to control the airflow. Anterior open bite can typically impact the production of sibilants due to the inability to create this narrow air passage; Leavy et al. [52] concluded that an open bite as little as 2 mm can impact the sound production of this phoneme group. The characteristic interdental positioning of the tongue during speech alters airflow, leading to noticeable distortions, especially lisping. The impact of AOB on sibilants has been confirmed by earlier studies [20,53], as well as studies included in this review [37,39,40,41,42]. Class II malocclusions can similarly impact sibilants due to the typical overjet of and the corresponding difficulty in performing a correct lip seal [20,54,55,56], confirmed by studies included in this review [37,39,41]. In subjects with Class III malocclusion, sibilant production is impacted by the characteristically low-positioned and retruded tongue posture at rest; this has been reported by prior literature [20,57] and studies included in this review [37,40,41]. Some individuals may develop compensatory articulation techniques by adapting the tongue position [22] or by mandibular movements [21], while others might have more pronounced speech difficulties. Given that each individual might develop unique compensatory mechanisms, understanding these can provide insights into personalized therapeutic approaches.

Despite the slight discrepancies in reporting, probably due to the differences between the phonemes and the malocclusions considered, the pronunciation problems reported for the sibilant sounds are broadly superimposable to those related to the pronunciation of the phonemes /t/, /d/, and /n/ by the articles included in this review [36,37,39,40,41,42]. This is due to the fact that they require a similar articulation with the tip of the tongue to touch the alveolar ridge, completely closing the air passage. Therefore, open bite, increased overjet, and low tongue position negatively impact the production of these sounds.

Not all studies included in this review detailed which phonemes are negatively affected by which malocclusions. The broad impact of anterior open bite and Class II and Class III malocclusions on a wide range of phonemes /s/, /z/, /ch/, /t/, /d/, and /n/ is somewhat confirmed by others, as subjects with these malocclusions were statistically more likely to suffer from phonation issues. The results presented by Mogren [32,33] and Farronato [31] agree on this and on the greater impact of anterior open bite and Class III malocclusions compared to Class II malocclusions. Contrarily, Sahad [36] reports a comparable impact between Class II and Class III malocclusions, and found AOB less impacting than the other studies included in this review. While the included studies do not explore different impacts on phonemes in different languages, the results summarized in Table 5 show how the more frequently reported phonemes (/s/, /z/, /t/, /d/, and /n/) in various languages: English [40], Spanish [41], Portuguese [37], and Polish [39] are impacted by the same malocclusions. This is in line with the observations made by Amr-Rey in [41].

This scoping review excluded studies which considered, by design or chance, patients with neurodevelopmental disorders and other syndromes or contributing medical conditions such as cleft palate, mandibular joint disorders, and Down syndrome. As discussed previously, function can impact structure, so explicitly excluding patients with medical conditions that might cause SSDs per se can increase the confidence that malocclusion causes speech sound disorders and not vice versa, but there is still disagreement in the literature, and a complex interplay of contributing factors needs to be considered.

On the other end, this review has the following limitations:

- The presence of articles with different granularities of investigation on the relation between SSDs and malocclusions;
- Each article considered a different set of malocclusions, with a clear skewing towards AOB;
- There was a mixture of languages in the selected population, as pronunciation defects might vary across populations and languages;
- Since this is a scoping review, a quality assessment of the included studies was not performed, and the heterogeneity of the study designs included was significant.

The identified gaps signal the need for longitudinal studies that track changes over time, providing insights into how malocclusions develop and evolve from early childhood through adolescence, and help establish whether the onset of certain malocclusions coincides with or predates the emergence of speech sound disorders.

The relation between some types of malocclusions and speech sound disorders is less explored. Lateral cross-bite is a notable example in some studies [20,41,46,58] that identified a relation with phonetic alterations, which can be explained by a lower resting position of the tongue, but still requires a more in-depth investigation to clarify the nature of this relation.

A general consensus has however been found by all the articles selected for this review on the multifactorial nature of SSDs and the need for a multidisciplinary approach for an effective treatment. This is also highlighted by Cenzato et al. [59] in a review on open bite and atypical swallowing treatment, noting that the most effective treatment is a combination of traditional orthodontic therapy and myofunctional logopedic therapy.

## 5. Conclusions

The relationship between malocclusions and speech sound disorders is well documented in the literature, indicating a significant correlation that is confirmed by all studies included in this scoping review.

The outcome of this scoping review highlights the following:

- Among various types of malocclusions, anterior open bite is particularly notable for its high frequency and pronounced impact on phoneme production.
- Class II and Class III malocclusions’ impact on speech production is similar and comparable to that of AOB.
- The phonemes /r/, /s/, /z/, /ch/, /f/, /d/, /t/, /n/, /l/, and /ȝ/ are found to be impacted by most malocclusions, signaling again the significant correlation between the two conditions.

The exact causal dynamics between malocclusions and SSDs remain elusive, however, and further research is needed, possibly through longitudinal studies, to provide conclusive evidence on these aspects. The complex interplay between these two conditions is probably a vicious cycle where one condition is influenced by the other and vice versa; this underlines the need for a multidisciplinary approach to treatment, integrating orthodontic, myofunctional, and logopedic therapies.

## Figures and Tables

**Table 1:** Search strategy.

Database	Search Strategy	Number of Results
The Cochrane Library	(“malocclusion” OR “anterior open bite” OR “open bite” OR “AOB” OR “anterior cross bite” OR “posterior cross bite” OR “cross bite” OR “deep bite”) AND (“speech disorder” OR “speech” OR “phoneme” OR “articulation disorder” OR “sound” OR “affricative” OR “fricative” OR “sibilant” OR “dyslalias” OR “affricate”) AND (“deciduous dentition” OR “primary dentition” OR “early mixed dentition” OR “late mixed dentition” OR “mixed dentition” OR “pediatric” OR “child” OR “children” OR “scholars”)	44
PubMed	(“malocclusion” OR “anterior open bite” OR “open bite” OR “AOB” OR “anterior cross bite” OR “posterior cross bite” OR “cross bite” OR “deep bite”) AND (“speech disorder” OR “speech” OR “phoneme” OR “articulation disorder” OR “sound” OR “affricative” OR “fricative” OR “sibilant” OR “dyslalias” OR “affricate”) AND (“deciduous dentition” OR “primary dentition” OR “early mixed dentition” OR “late mixed dentition” OR “mixed dentition” OR “pediatric” OR “child” OR “children” OR “scholars”)	505
Scopus	(“malocclusion” OR “anterior open bite” OR “open bite” OR “AOB” OR “anterior cross bite” OR “posterior cross bite” OR “cross bite” OR “deep bite”) AND (“speech disorder” OR “speech” OR “phoneme” OR “articulation disorder” OR “sound” OR “affricative” OR “fricative” OR “sibilant” OR “dyslalias” OR “affricate”) AND (“deciduous dentition” OR “primary dentition” OR “early mixed dentition” OR “late mixed dentition” OR “mixed dentition” OR “pediatric” OR “child” OR “children” OR “scholars”)	696
Web of Science	(“malocclusion” OR “anterior open bite” OR “open bite” OR “AOB” OR “anterior cross bite” OR “posterior cross bite” OR “cross bite” OR “deep bite”) AND (“speech disorder” OR “speech” OR “phoneme” OR “articulation disorder” OR “sound” OR “affricative” OR “fricative” OR “sibilant” OR “dyslalias” OR “affricate”) AND (“deciduous dentition” OR “primary dentition” OR “early mixed dentition” OR “late mixed dentition” OR “mixed dentition” OR “pediatric” OR “child” OR “children” OR “scholars”)	635

**Table 2:** Inclusion and exclusion criteria.

Inclusion	Exclusion
Study design: case–control studies, cross-sectional studies, cohort studies, case reports, and case reports series	No articles about treatment of malocclusion and speech disorders
Type of publication: articles published in journals with impact factor or cited by articles published in journals with impact factor	No articles about other medical conditions such as cleft palate, tempo mandibular joint disorders, idiopathic juvenile arthritis, Down syndrome, etc.
Participants: patients in the developmental stage of dentition, i.e., deciduous dentition (up to 6 years) or mixed dentition (6–12 years)	The article is not a review or a letter to the editor
Only articles about correlation between malocclusion and speech disorders	

**Table 5:** Relation between each malocclusion and affected phonemes as reported by each study.

Phoneme	AOB	Class I	Class II	Class III	Cross-Bite	Edge-to-Edge Bite
/r/		[37]	[37,41]	[37,41]	[37]	[41]
/rr/			[41]	[41]		
/s/	[37,39,40,41,42]	[37]	[37,39,41]	[37,40,41]	[37,41]	[41]
/z/	[37,39,40,41]	[37]	[37,39,41]	[37,40,41]	[37,41]	[41]
/sh/	[41]					
/zh/	[41]					
/f/	[37,41]		[41]	[37]	[37,41]	
/v/	[37,41]			[37]	[37]	
/ch/	[41,42]		[41]	[41]	[41]	
/ll/						[41]
/th/	[41]					
/t/	[36,40,41,42]	[37]	[39]	[37,39,40]	[37,41]	[37,41]
/d/	[36,39,40,41,42]	[37]	[39,40]	[37,39,40]	[41]	
/n/	[36,39,40,41]	[37]	[39]	[37,39]	[41]	
/l/	[36,41]	[37]	[40]	[37,40,41]		[41]
/ɲ/	[41,42]					
/ȝ/	[37]	[37]	[37]	[37]	[37]	
/ɕ/			[39]	[39]		
/ʑ/			[39]	[39]		
/t͡s/	[39]		[39]	[39]		
/d͡z/	[39]		[39]	[39]		
/t͡ɕ/	[39]		[39]	[39]		
/d͡ʑ/	[39]		[39]	[39]		

**Table 6:** Reference table for the terms used throughout this review to categorize phonemes and sounds referenced in the selected studies.

Phonemes	Articulation Type	Articulation Place	Acoustic
/r/, /rr/	Trill	Linguo-alveolar	Rhotic sounds
/s/, /z/	Fricative	Linguo-alveolar	Narrow constriction at the alveolar ridge causing turbulent airflow. Frequency is high for sibilants (/s/, /z/, /sh/, /zh/, /ȝ/) and low for non-sibilants
/sh/, /zh/, /ȝ/	Fricative	Post-alveolar	
/f/, /v/	Fricative	Labiodental	
/ɕ/, /ʑ/	Fricative	Alveo-palatal	
/th/	Fricative	Dental	
/t/, /d/	Plosive	Linguo-alveolar	Complete closure at the alveolar ridge, followed by a burst of air
/l/	Lateral approximant	Linguo-alveolar	Rapid vibration of articulators with airflow passing on the tongue sides
/ll/	Lateral approximant	Palatal	Smooth airflow passing on the tongue sides
/n/	Nasal	Linguo-alveolar	Airflow passes through the nasal cavity
/ch/	Affricate	Post-alveolar	Affricates are pronounced as a plosive followed by a fricative
/t͡s/, /d͡z/	Affricate	Linguo-alveolar	
/t͡ɕ/, /d͡ʑ/	Affricate	Alveolo-palatal	

## Data Availability

The raw data supporting the conclusions of this article will be made available by the authors upon request.
